# Population Responses during the Pandemic Phase of the Influenza A(H1N1)pdm09 Epidemic, Hong Kong, China

**DOI:** 10.3201/eid2305.160768

**Published:** 2017-05

**Authors:** Nelson C.Y. Yeung, Joseph T.F. Lau, Kai Chow Choi, Sian Griffiths

**Affiliations:** The Chinese University of Hong Kong, The Jockey Club School of Public Health and Primary Care, Hong Kong, China (N.C.Y. Yeung, J.T.F. Lau, S. Griffiths);; The Chinese University of Hong Kong, The Nethersole School of Nursing, Hong Kong (K.C. Choi)

**Keywords:** influenza A(H1N1)pdm09 virus, H1N1, pandemic, influenza, swine flu, population responses, behavioral responses, psychological responses, perceptions, Hong Kong, China, epidemic, epidemic, outbreak, viruses, longitudinal survey, respiratory infections

## Abstract

During August 2009–July 2010, we conducted 7 longitudinal telephone surveys among 503 adults in Hong Kong, China, to explore changes in their behavioral and psychological responses to the influenza A(H1N1)pdm09 virus epidemic. Trends were examined using generalized estimating equations models. Findings showed that responses varied with the course of the pandemic.

On June 11, 2009, the World Health Organization declared the influenza A(H1N1)pdm09 (pH1N1) virus outbreak a pandemic (*1*). Previous studies have investigated community responses to the pandemic in different countries during early stages of the epidemic (*2*–*5*). The studies investigated persons’ risk perceptions and knowledge related to the virus, perceived efficacy of preventive measures, and psychological and behavioral responses. However, because of intersample variations, these cross-sectional studies did not capture within-person changes. We conducted a longitudinal cohort study to investigate changes in responses among the general Hong Kong, China, population during the pH1N1 pandemic.

## The Study

A cohort sample of 18- to 60-year-old adults in Hong Kong participated in 7 rounds of telephone surveys during August 2009–July 2010, which covered almost the entire pH1N1 pandemic period in Hong Kong. At baseline, we invited 677 adults to participate; 503 (74.3%) consented and completed the survey (Technical Appendix Table 1). We measured the following variables: knowledge about the modes of pH1N1 virus transmission; risk perceptions associated with the virus (perceived susceptibility to and severity of infection); perceived efficacy and use of preventive measures (e.g., handwashing, using a facemask); psychological responses (worry about infection and emotional distress); and evaluations of the government’s performance in pandemic control. Sample sizes for surveys 2–7 ranged from 452 to 481, yielding retention rates of 89.8%–95.6%.

Most participants were women (57.9%), 40–60 years of age (55.8%), employed full time (55.9%), and married (65%). Sex and age distributions were comparable to those in the local census data (*6*). We aimed to determine whether there were overall linear trends in participants’ perceptions, psychological responses, and behavioral responses to the pandemic. We examined linear trends for these variables across the 7 time points by using generalized estimating equations (GEE) models. GEE models not only account for intracorrelated repeated measures data but also fit various data types using appropriate link functions. The analyses were conducted using PROC GENMOD (SAS Institute, Cary, NC, USA); 2-sided p<0.05 was considered significant.

Over time, >85% of the participants used a face mask and immediately visited a doctor when experiencing influenza-like symptoms. More than 50% of the participants washed their hands >10 times every day throughout the survey period (p>0.05). As the pandemic progressed, a decreasing percentage of participants wore masks in public areas; avoided touching their mouth, nose, and eyes; took antiviral drugs; and avoided crowded places (p<0.001) (Technical Appendix Table 2). Percentages of participants feeling worried, depressed, or emotionally disturbed about pH1N1 virus decreased over time (p<0.001) (Technical Appendix Table 3).

Over time, a decreasing percentage of participants recognized that touching infected persons or contaminated objects could result in virus transmission (p<0.001). Throughout the study period, a consistently high percentage of participants (>92%) recognized that the virus could be transmitted via respiratory droplets. Misconceptions about possible transmission through insect bites (26.1%) and water sources (34.5%) were prevalent. The percentage of participants reporting at least 1 misconception was stable over time (p>0.05). A consistently high percentage (>90%) of participants believed that using face masks in public areas, washing hands frequently, and avoiding crowded places could effectively prevent the spread of pH1N1 virus (p>0.05).

The percentage of participants believing that pH1N1 virus would be more harmful than seasonal influenza in terms of fatality and bodily damage increased over time (p<0.001). The percentages of participants who believed the population was highly susceptible to pH1N1virus infection and who perceived a high chance of having a large-scale local outbreak in the coming year dropped significantly (p<0.001), but some fluctuations were observed; for example, the percentage peaked during survey round 2 (around the September influenza season).

Throughout the study period, ≈12%–21% of the participants gave a failing score (<5 on a 0- to 10-point scale) for the government’s overall performance in controlling the pandemic (p>0.05). However, during survey rounds 2–7, an increasing percentage of participants believed in the government’s ability to control the pandemic (p<0.001) (online Technical Appendix). The percentage of participants who believed that Hong Kong would not have enough vaccine or medication to deal with the pandemic decreased over time (p<0.001).

## Conclusions

This study investigated changes in community perceptions over the course of the pH1N1 pandemic in Hong Kong. Findings were highly comparable to those from other local cross-sectional surveys (*5*,*7*) and a systematic review (*8*). Knowledge regarding preventive measures and adherence to such measures was, in general, higher among our participants than among the general population in other countries (e.g., Australia, India, and the Netherlands) (*9*–*11*). The prevalence of misconceptions about some incorrect modes of transmission (e.g., insect bites) gradually declined. However, ≈50% of participants still held at least 1 of the 4 misconceptions regarding transmission (i.e., airborne transmission over a long distance and transmission through insect bites, water sources, and well-cooked pork). Furthermore, over time, a lower percentage of participants avoided touching their eyes, nose, and mouth to prevent virus transmission. A 2015 systematic review suggested that health authorities should provide more updated information about the virus (*8*). We also recommend using health campaigns to increase public awareness about different routes of pH1N1 virus transmission.

Perceived severity of pH1N1 virus infection decreased over time, which may partially explain the decline in distress and avoidance behaviors; this pattern was also observed in a recent review (*8*). However, an increasing proportion of participants believed that, compared with seasonal influenza, pH1N1 resulted in more deaths and more severe body damage. Perceived susceptibility to infection declined substantially as the epidemic progressed, suggesting that the public gradually perceived fewer risks from pH1N1 virus. Avoidance behaviors and use of facemasks in the absence of influenza-like symptoms became less prevalent over time, similar to a trend seen in Malaysia (*12*). Mental distress among persons in Hong Kong was lower during the pH1N1 pandemic than during the SARS (severe acute respiratory syndrome) pandemic (*13*), possibly due to the milder consequences of pH1N1 infection. Persons in Hong Kong seemed to remain rational during the pandemic, thereby avoiding possible pandemic-associated economic threats.

Public support for the government declined over time. During survey round 5, a total of 20.6% of the participants gave a failing score to the government’s performance, and 13.5% perceived that the government would not be able to control the pandemic. The poll was split as to whether the government should use the same response for pH1N1 influenza and seasonal influenza. Our findings suggest that the public should be advised of the pros and cons of pH1N1 control policies; a watchful step-down may be better accepted if the policies are understood.

This study has limitations. First, telephone surveys may be subject to self-selection bias. However, participants’ demographics were comparable to those in local census data (*6*). Second, Hong Kong’s unique experience with the SARS outbreak may have influenced the population’s response to the pH1N1 pandemic; thus, our findings may not be fully generalizable to other countries. Third, we treated time as a continuous variable in the GEE models. Ideally, polynomials should be added to the linear time variable; however, given the small number of time points and absence of theoretical shapes, that was not feasible.

Our findings provide valuable information regarding overall linear trends and changes in community responses toward the pH1N1 pandemic among a Hong Kong cohort. These findings should help inform other countries in formulating appropriate pandemic control plans for influenza and other emerging infectious diseases.

Technical Appendix Detailed methodology and findings for a study of population responses during the pandemic phase of the influenza A(H1N1)pdm09 epidemic, Hong Kong, China.

## References

[1] World Health Organization. World now at the start of 2009 influenza pandemic. Statement to the press by WHO Director-General Dr. Margaret Chan. June 11, 2009 [cited 2017 Mar 9]. http://www.who.int/mediacentre/news/statements/2009/h1n1_pandemic_phase6_20090611/en/

[2] Lau JT, Griffiths S, Choi KC, Tsui HY. Widespread public misconception in the early phase of the H1N1 influenza epidemic. J Infect. 2009;59:122–7. 10.1016/j.jinf.2009.06.004 19592114

[3] Rubin GJ, Amlôt R, Page L, Wessely S. Public perceptions, anxiety, and behaviour change in relation to the swine flu outbreak: cross sectional telephone survey. BMJ. 2009;339(jul02 3):b2651. 10.1136/bmj.b2651 PMC2714687 19574308

[4] Effler PV, Carcione D, Giele C, Dowse GK, Goggin L, Mak DB. Household responses to pandemic (H1N1) 2009-related school closures, Perth, Western Australia. Emerg Infect Dis. 2010;16:205–11. 10.3201/eid1602.091372 20113548 PMC2958027

[5] Cowling BJ, Ng DMW, Ip DKM, Liao Q, Lam WWT, Wu JT, et al. Community psychological and behavioral responses through the first wave of the 2009 influenza A(H1N1) pandemic in Hong Kong. J Infect Dis. 2010;202:867–76. 10.1086/655811 20677945

[6] Census and Statistics Department, Hong Kong SAR Government. Digest of Statistics, 2009 edition [cited 2017 Mar 9]. http://www.statistics.gov.hk/pub/B10100032009AN09B0700.pdf

[7] Liao Q, Cowling BJ, Lam WW, Ng DMW, Fielding R. Anxiety, worry and cognitive risk estimate in relation to protective behaviors during the 2009 influenza A/H1N1 pandemic in Hong Kong: ten cross-sectional surveys. BMC Infect Dis. 2014;14:169. 10.1186/1471-2334-14-169 24674239 PMC3986671

[8] Bults M, Beaujean DJMA, Richardus JH, Voeten HACM. Perceptions and behavioral responses of the general public during the 2009 influenza A (H1N1) pandemic: a systematic review. Disaster Med Public Health Prep. 2015;9:207–19. 10.1017/dmp.2014.160 25882127

[9] Seale H, McLaws ML, Heywood AE, Ward KF, Lowbridge CP, Van D, et al. The community’s attitude towards swine flu and pandemic influenza. Med J Aust. 2009;191:267–9. 19740048 10.5694/j.1326-5377.2009.tb02781.x

[10] Kamate SK, Agrawal A, Chaudhary H, Singh K, Mishra P, Asawa K. Public knowledge, attitude and behavioural changes in an Indian population during the Influenza A (H1N1) outbreak. J Infect Dev Ctries. 2009;4:7–14. 20130372 10.3855/jidc.501

[11] Bults M, Beaujean DJMA, de Zwart O, Kok G, van Empelen P, van Steenbergen JE, et al. Perceived risk, anxiety, and behavioural responses of the general public during the early phase of the Influenza A (H1N1) pandemic in the Netherlands: results of three consecutive online surveys. BMC Public Health. 2011;11:2. 10.1186/1471-2458-11-2 21199571 PMC3091536

[12] Lau JTF, Griffiths S, Choi KC, Lin C. Prevalence of preventive behaviors and associated factors during early phase of the H1N1 influenza epidemic. Am J Infect Control. 2010;38:374–80. 10.1016/j.ajic.2010.03.002 20569849 PMC7132693

[13] Lau JTF, Griffiths S, Choi KC, Tsui HY. Avoidance behaviors and negative psychological responses in the general population in the initial stage of the H1N1 pandemic in Hong Kong. BMC Infect Dis. 2010;10:139. 10.1186/1471-2334-10-139 20509887 PMC2891756

