# Supplementary material for: Population Responses during the Pandemic Phase of the Influenza A(H1N1)pdm09 Epidemic, Hong Kong, China
Source: Emerg Infect Dis. 2017 May;23(5):813–5. doi: 10.3201/eid2305.160768 (PMC5403031; doi:10.3201/eid2305.160768)
Supplement: Technical Appendix — Detailed methodology and findings for a study of population responses during the pandemic phase of the influenza A(H1N1)pdm09 epidemic, Hong Kong, China. [file 16-0768-Techapp-s1.pdf]

Article DOI: http://dx.doi.org/10.3201/eid2305.160768

Population Responses during the Pandemic Phase of the Influenza A(H1N1)pdm09 Epidemic, Hong Kong, China

Technical Appendix

Introduction

Previous findings on community responses at different phases of the H1N1 epidemic

During the pre-community outbreak phase of the H1N1 epidemic from May 6 to June 6 2009, two studies in Hong Kong reported a moderate level of perceived susceptibility of contracting the disease (around 7.5% to 12.1% of the general population felt susceptible) and perceived severity of H1N1 (over 15% of general population believed that H1N1 is highly fatal), plus a low level of H1N1-related distress in the community (less than 5% were panicking). Misconceptions about the modes of H1N1 transmissions were common. These studies found that perceptions related to bodily damages, efficacy of frequent handwashing, non-availability of effective vaccines, chance of having a large-scale local outbreak, and mental distress due to influenza A/H1N1 were associated with frequent handwashing (*1*,*2*). Another study involving a series of cross-sectional surveys was also conducted, suggesting a generally low level of anxiety and a slight improvement in knowledge on modes of transmission throughout the epidemic. The perceived susceptibility and perceived severity of getting H1N1 were high in the early phase, but started to decline in the early pandemic phase epidemic and remained stable thereafter. Preventive behaviors like handwashing and wearing facemasks did not show significant change throughout the epidemic, while avoidance behaviors like avoiding going to crowded places declined gradually at the pandemic unfolds (*3*). During the early phase of the global H1N1 pandemic, other international studies reported a moderate level of distress in the U.S., a lack of preventive responses such as change in frequency of handwashing in the UK (*4*) and in India, plus a lack of support

for governmental mitigation strategies, such as school closure in Australia (*5*). Other studies found that Korean college students and Italian healthcare workers washed their hands or used hand sanitizer more frequently than before the beginning of the pandemic. The Chinese general population in seven urban regions and two rural regions showed an increasing level of knowledge, but a declining level of risk perception and adoption of preventive measures, while the U.S. general population showed an increasing trend in perceived risk but a decreasing trend in prevalence of precautionary activities (*6*). A study in Malaysia also showed that preventive measures, fear and avoidance changed in concordance with the trend in number of reported deaths (*7*).

Methods

The specific dates for the seven rounds of surveys

The study population comprised of Chinese adults (aged 18–60) who were living in Hong Kong. Seven longitudinal telephone surveys were conducted during August 2009 through July 2010 (Round 1: Aug 19 – Sep 10; Round 2: Sep 25 – Oct 5; Round 3: Oct 27 – Nov 2; Round 4: Nov 28 – Dec 10; Round 5: Jan 28 – Feb 5; Round 6: Mar 24 – Apr 1; Round 7: Jun 24 – Jul 10). During the study period, the Hong Kong Hospital Authority lowered the influenza response level from "Emergency" to "Alert" Response Level on May 24, 2010. The numbers of H1N1 death cases at the end of the seven survey dates were respectively 4, 20, 37, 41, 64, 76 and 80.

Details of study procedure

At least three other independent telephone calls were made at different hours/days to unanswered telephone numbers. Over 95% of the households in Hong Kong have a fixed-line telephone installed (*5*). To avoid over-representing people who were not employed, the baseline survey was conducted during 6:30–10 p.m. Verbal consent was sought from the respondents before the interview commenced. Participants provided us with their name (or nickname) for identification, at least two contact phone numbers, and time that they were usually available for phone interview. Participants were given a supermarket voucher (HKD$50 or about USD$6.5) by mail as compensation for the completion of each survey.

Results

Background characteristics

The gender/age distributions were comparable to those of the recent census data (% female = 46.0%; % 40–60 = 51.5%). Proportion of female participants was higher among the respondents than among the non-responders (57.9% versus 45.4%, $p < 0.01$). No significant between-group differences were found for other socio-demographic characteristics ($p > 0.05$; Table 1).

Preventive and avoidance behaviors

Over time, >85% of the participants used a face mask and immediately visited a doctor when experiencing influenza-like symptoms. More than 50% of the participants washed their hands >10 times every day throughout the survey period ($p>0.05$). As the pandemic progressed, a decreasing percentage of participants wore masks in public areas; avoided touching their mouth, nose, and eyes; took anti-influenza drugs; avoided crowded places; and took Traditional Chinese medicine to prevent virus infection ($p<0.01$) (Table 2).

Worry and distress related to H1N1

Fewer participants worried about themselves (from 12.3% in Round 1 to 2.4% in Round 7, $p < 0.001$) or their family members (from 15.3% in Round 1 to 2.4% in Round 7, $p < 0.001$) contracting H1N1, while more participants believed that "it does not matter if I contracted H1N1' (from 41.4% in Round 1 to 50.7% in Round 7, $p<.001$). Fewer participants were in panic, feeling much depressed or felt emotionally disturbed due to H1N1, the prevalence at Round 7 became <1% for these three individual items. Percentage of participants expressing any type of mental distress (feeling panic/depressed/emotionally disturbed about H1N1) also significantly decreased from Round 1 to 7 (from 16.3% to 2.8%. $p<.001$) (Table 3).

Knowledge regarding modes of transmission of H1N1

Over time, a decreasing percentage of participants recognized that touching infected persons and touching contaminated objects could transmit H1N1 ($p < 0.001$). Throughout the whole study period, a consistently high percentage of participants (>92%)

recognized that H1N1 could be transmitted through droplets. Misconceptions about possible transmissions of H1N1 via insect bites (26.1%) and water sources (34.5%) were prevalent throughout the study period (Table 4).

Perceived severity of H1N1

Over time, fewer participants had mistaken that fatality rate of H1N1 was ≥1% (Round 1:31.4% to Round 7: 24.8%, such percentage peaked at Round 5: 47.6%) (Table 5).

Perceived susceptibility of H1N1

Fewer participants perceived a chance of having a large-scale local outbreak in the coming year (from 41.4% in Round 1 to 3.4% in Round 7, Table 6, $p < 0.001$). Similar percentages of respondents were confident in keeping themselves and their family free from H1N1 over time (over 70% at all rounds and peaking at Round 7: 92.3%, Table 6). Nevertheless, the prevalence of perceived susceptibility in contracting H1N1 fluctuated over time, in terms of susceptibility for oneself (Round 1:16.6% to Round 7: 2.6%), one’s family (Round 1: 14.9% to Round 7: 2.6%) and the general public (Round 1: 20.9% to Round 7: 2.6%) (Table 6).

Perceived efficacy of preventive measures

A consistently high percentage (>90%) of the participants believed that using face masks in public areas, washing hands frequently, and avoiding crowded places could effectively prevent the spread of H1N1 ($p > 0.05$) (Table 7).

Support toward government and pandemic control policies

Less than 10% of the participants at all rounds believed that the Hong Kong government overreacted to the H1N1 pandemic ($p > 0.05$; Table 8). Less than 5% of the participants at Rounds 2–6 believed that the government should cancel all control measures for controlling the H1N1 pandemic, but the percentage peaked at Round 7 (15.6%; Table 9). During Round 2 through 7, more participants believed in government’s ability to control the H1N1 pandemic (from 77.1% in Round 1 to 94.9% in Round 7, $p < 0.001$; Table 9), while a sizable proportion of participants believed that the government should treat H1N1 in the same manner as seasonal flu fluctuated (R1: 53.1%, R5: 38.9%, R7: 54.4%, $p < 0.001$) (Table 9).

Discussion

Comparison between Hong Kong community responses and those in other countries

Our participants had in general higher levels of knowledge (e.g., >92% recognizing transmission via droplets) and practice of preventive measures, as compared to general populations of other countries. For instance, only three-fourth of general population in China knew that H1N1 could be transmitted by droplets; less than 10% of the military men in Singapore were aware that influenza can be spread by touch (*8*); less than 25% of Indians and 50% of Australians believed that handwashing was effective in preventing H1N1; only one-fourth of general population in the Netherlands regarded wearing facemasks as an effective preventive measure and only 38% used preventive measures (*9*); only 6.5% of Korean college students reported washing their hands more than 10 times every day during the pandemic period.

Similarly, a study conducted in October 2009 showed that over 40% of medical students in Pakistan believed that avoiding pork consumption could effectively prevent H1N1. Another study in China showed that 30% of Chinese general population believed that food could transmit H1N1. In general, the general public in Hong Kong over-estimated the fatality rate of the H1N1 (around 40%) and believed that H1N1 is associated with higher fatality and bodily damages as compared to seasonal flu (50%–60%).

We confirmed that unlike SARS, mental distress is relatively light during the H1N1 pandemic, possible due to the milder consequences of H1N1 as compared to SARS. Except for the peak of incidence of H1N1 in September, only ≈10% of the respondents felt worried about contracting H1N1 and very few reported distress. About two-fifth of the general public expressed would not be bothered by H1N1 infection. No panic has been caused by the pandemic. Avoidance behaviors and use of face mask in the absence of ILI symptoms became less prevalent over time. Such a trend was also understandable as a Malaysian study showed that fear and avoidance changed according to the number of reported deaths (*7*). The general public in Hong Kong has apparently been becoming more rational to live with the pandemic, saving potential economic threats.

In contrast, decreasing trends in perceived severity of and anxiety associated with H1N1 were observed among Dutch population as the pandemic progressed (*9*). Perceived susceptibility was quite low and did not show obvious trend during the study period; 10%–15% of the general public considered himself/herself susceptible of contracting H1N1 in the coming year. Such findings are comparable to those in a study in Korea, where only 8% of the college students perceived high susceptibility to contracting H1N1.

As the pandemic progressed, we see a gradual increase in the proportions of participants believing that 1) school suspension policies is not necessary, 2) the government should suspend all pandemic control measures, and 3) the government should treat H1N1 as the same way as seasonal flu. At the same time, people were less worried about contracting H1N1, less likely to feel mental health distress, and engaging in preventive behaviors less frequently over time. It is potentially attributed to people's gaining understanding about the severity of the pandemic and getting used to the pandemic over time. These findings provide some support to the idea that people's risk perceptions of the disease and behavioral responses are consistent with their perceptions about the necessity of some pandemic control policies.

Governments in other countries seemed not to be well-supported. A study in Australia showed that people were not supporting the school closure policy as they believed that H1N1 was mild and the policy could not stop H1N1 from spreading (*5*). Another study in Turkey showed that the public generally perceived that its government was not managing H1N1 well and had a low level of trust to government's H1N1 information. A similar trend was also observed in the Netherlands, perceived reliability of government information decreased (*9*). Over half of Dutch respondents believed that the threat was exaggerated by the media and government (*9*).

Summary of findings

The general public maintains high level of preventive behaviors and a low level of mental distress throughout the study period. Compared to other countries, the general public in Hong Kong seemed to be more compliant to preventive behaviors like frequent handwashing and be more equipped with knowledge related to H1N1. Misconceptions became less prevalent but were still noticeable. Given the prior experience with SARS, Hong Kong government kept its vigilance for H1N1 by daily reporting of infection cases and frequent TV/radio commercials about H1N1 prevention. Dissemination of knowledge and frequent updates about H1N1 pandemic

from the government may partially explain the consistently high rate of preventive behaviors and relatively better knowledge. Perceived severity and susceptibility remained relatively stable and somehow low. The public rated the government very highly at the beginning at the pre-community outbreak phase (*1*) but the rating declined over time. However, the public was still supporting the government to have treat H1N1 differently from seasonal flu, and the overall support was still very reasonable.

References

1. Lau JT, Griffiths S, Choi KC, Tsui HY. Widespread public misconception in the early phase of the H1N1 influenza epidemic. J Infect. 2009;59:122–7. PubMed http://dx.doi.org/10.1016/j.jinf.2009.06.004

2. Lau JTF, Griffiths S, Choi KC, Lin C. Prevalence of preventive behaviors and associated factors during early phase of the H1N1 influenza epidemic. Am J Infect Control. 2010;38:374–80. PubMed http://dx.doi.org/10.1016/j.ajic.2010.03.002

3. Cowling BJ, Ng DMW, Ip DKM, Liao Q, Lam WWT, Wu JT, et al. Community psychological and behavioral responses through the first wave of the 2009 influenza A(H1N1) pandemic in Hong Kong. J Infect Dis. 2010;202:867–76. PubMed http://dx.doi.org/10.1086/655811

4. Rubin GJ, Amlôt R, Page L, Wessely S. Public perceptions, anxiety, and behaviour change in relation to the swine flu outbreak: cross sectional telephone survey. BMJ. 2009;339(jul02 3):b2651. PubMed http://dx.doi.org/10.1136/bmj.b2651

5. Effler PV, Carcione D, Giele C, Dowse GK, Goggin L, Mak DB. Household responses to pandemic (H1N1) 2009-related school closures, Perth, Western Australia. Emerg Infect Dis. 2010;16:205–11. PubMed http://dx.doi.org/10.3201/eid1602.091372

6. Ibuka Y, Chapman GB, Meyers LA, Li M, Galvani AP. The dynamics of risk perceptions and precautionary behavior in response to 2009 (H1N1) pandemic influenza. BMC Infect Dis. 2010;10:296. PubMed http://dx.doi.org/10.1186/1471-2334-10-296

7. Wong LP, Sam IC. Temporal changes in psychobehavioral responses during the 2009 H1N1 influenza pandemic. Prev Med. 2010;51:92–3. PubMed http://dx.doi.org/10.1016/j.ypmed.2010.04.010

8. Yap J, Lee VJ, Yau TY, Ng TP, Tor PC. Knowledge, attitudes and practices towards pandemic influenza among cases, close contacts, and healthcare workers in tropical Singapore: a cross-sectional survey. BMC Public Health. 2010;10:442. PubMed http://dx.doi.org/10.1186/1471-2458-10-442

9. Bults M, Beaujean DJ, de Zwart O, Kok G, van Empelen P, van Steenbergen JE, et al. Perceived risk, anxiety, and behavioural responses of the general public during the early phase of the Influenza A (H1N1) pandemic in the Netherlands: results of three consecutive online surveys. BMC Public Health. 2011;11:2. PubMed http://dx.doi.org/10.1186/1471-2458-11-2

Technical Appendix Table 1. Background characteristics, Census & Statistics Department HKSAR, 2009*

Characteristic	n = 503	%
Gender		
Male	212	42.1
Female	291	57.9
Age†‡		
<30	122	24.3
30 – 39	100	19.9
40 – 49	154	30.7
50 – 60	126	25.1
Education level		
Form 3 or below	79	15.8
Form 4 – matriculation	217	43.3
College or above	205	40.9
Marital status		
Single	168	33.4
Married /cohabited	327	65.0
Divorced /widowed	8	1.6
Employment status		
Full-time	280	55.9
Part-time	18	3.6
Students	46	9.2
Retired	25	5.0
Housewife	113	22.6
Unemployed	18	3.6
Others	1	0.2
Current health care practitioner		
No	482	97.4
Yes	13	2.6

*Based on the census data published in Hong Kong Annual Digest of Statistics, 2009
†the proportions of male and female of age 18–60 in Hong Kong were respectively
46.02% and 53.98%
‡the proportions of the 4 age groups in Hong Kong (<30, 30–39, 40–49, and 50–60) were respectively 24.75%, 23.78%, 27.33%, and 24.14%.

Technical Appendix Table 2. Preventive and avoidance behaviors related to H1N1 influenza A/H1N1

Preventive or avoidance behavior	Round 1 (n = 503), 19 Aug – 10 Sep 09	Round 2 (n = 481), 25 Sep – 5 Oct 09	Round 3 (n = 475), 27 Oct – 2 Nov 09	Round 4 (n = 473), 28 Nov – 10 Dec 09	Round 5 (n = 452), 28 Jan – 5 Feb 10	Round 6 (n = 457), 7 – 14 Apr 10	Round 7 (n = 467), 24 Jun – 6 Jul 10	p (trend)
Preventive behaviors								
Wearing mask when going out in case of ILI symptoms								
Most not / certainly not / unsure	37 (7.4%)	51 (10.6%)	58 (12.2%)	70 (14.8%)	45 (10.0%)	58 (12.7%)	63 (13.5%)	0.012
Mostly /certainly	466 (92.6%)	430 (89.4%)	416 (87.8%)	403 (85.2%)	407 (90.0%)	398 (87.3%)	404 (86.5%)	
Consult a doctor immediately if you have fever								
Most not / certainly not / unsure	39 (7.8%)	22 (4.6%)	13 (2.7%)	22 (4.7%)	38 (8.4%)	49 (10.7%)	56 (12.0%)	<0.001
Mostly /certainly	464 (92.2%)	459 (95.4%)	462 (97.3%)	451 (95.3%)	414 (91.6%)	408 (89.3%)	411 (88.0%)	

Preventive or avoidance behavior	Round 1 (n = 503), 19 Aug – 10 Sep 09	Round 2 (n = 481), 25 Sep – 5 Oct 09	Round 3 (n = 475), 27 Oct – 2 Nov 09	Round 4 (n = 473), 28 Nov – 10 Dec 09	Round 5 (n = 452), 28 Jan – 5 Feb 10	Round 6 (n = 457), 7 – 14 Apr 10	Round 7 (n = 467), 24 Jun – 6 Jul 10	p (trend)
Tell immigration control if you have the flu or cold-like symptoms								
Most not / certainly not / unsure	23 (4.6%)	17 (3.5%)	8 (1.7%)	16 (3.4%)	–	–	–	0.099
Mostly /certainly	480 (95.4%)	464 (96.5%)	466 (98.3%)	457 (96.6%)	–	–	–	
Wearing mask in public areas if you have not IL symptoms in the last week								
No / Rarely	435 (86.5%)	399 (83.5%)	436 (91.8%)	438 (92.8%)	417 (92.7%)	436 (95.4%)	452 (96.8%)	<0.001
Sometimes / Always	68 (13.5%)	79 (16.5%)	39 (8.2%)	34 (7.2%)	33 (7.3%)	21 (4.6%)	15 (3.2%)	
Frequency of washing hands per day								
1 – 10	253 (50.4%)	229 (47.6%)	203 (42.7%)	206 (43.6%)	213 (47.2%)	221 (48.4%)	197 (42.3%)	0.075
>10	249 (49.6%)	252 (52.4%)	272 (57.3%)	267 (56.4%)	238 (52.8%)	236 (51.6%)	269 (57.7%)	
Avoid touching mouth, nose and eyes so as to reduce the risk of contracting Flu A (H1N1)								
No / Rarely	326 (64.8%)	311 (64.9%)	371 (78.1%)	371 (78.6%)	318 (70.8%)	347 (76.3%)	341 (73.0%)	0.003
Sometimes / Always	177 (35.2%)	168 (35.1%)	104 (21.9%)	101 (21.4%)	131 (29.2%)	108 (23.7%)	126 (27.0%)	
Take Tamiflu or other anti-flu drugs								
Most not / certainly not / unsure	468 (93.6%)	463 (97.9%)	470 (99.2%)	456 (97.2%)	445 (98.5%)	454 (99.6%)	461 (98.7%)	<0.001
Mostly /certainly	32 (6.4%)	10 (2.1%)	4 (0.8%)	13 (2.8%)	7 (1.5%)	2 (0.4%)	6 (1.3%)	
Take Traditional Chinese Medicine to prevent human swine flu								
Most not / certainly not / unsure	455 (90.8%)	442 (93.6%)	441 (93.0%)	438 (93.4%)	416 (92.0%)	430 (94.3%)	443 (94.9%)	0.009
Mostly / certainly	46 (9.2%)	30 (6.4%)	33 (7.0%)	31 (6.6%)	36 (8.0%)	26 (5.7%)	24 (5.1%)	
Avoidance behaviors								
Avoid going to crowed place								
Most not / certainly not / unsure	223 (44.3%)	227 (47.3%)	282 (59.4%)	284 (60.0%)	274 (60.6%)	298 (65.2%)	355 (76.0%)	<0.001
Mostly / Certainly	280 (55.7%)	253 (52.7%)	193 (40.6%)	189 (40.0%)	178 (39.4%)	159 (34.8%)	112 (24.0%)	
Avoid going out unless it is necessary								
Most not / certainly not / unsure	296 (58.8%)	319 (66.3%)	374 (78.7%)	362 (76.5%)	354 (78.3%)	351 (76.8%)	373 (79.9%)	<0.001
Mostly / Certainly	207 (41.2%)	162 (33.7%)	101 (21.3%)	111 (23.5%)	98 (21.7%)	106 (23.2%)	94 (20.1%)	
Avoid traveling abroad								
Most not / certainly not / unsure	262 (52.2%)	286 (59.5%)	330 (69.5%)	309 (65.3%)	320 (70.8%)	337 (73.7%)	373 (79.9%)	<0.001
Mostly / Certainly	240 (47.8%)	195 (40.5%)	145 (30.5%)	164 (34.7%)	132 (29.2%)	120 (26.3%)	94 (20.1%)	
Avoid going to hospitals								
Most not / certainly not / unsure	222 (44.3%)	206 (43.0%)	266 (56.0%)	256 (54.1%)	268 (59.3%)	306 (67.0%)	317 (67.9%)	<0.001
Mostly / Certainly	279 (55.7%)	273 (57.0%)	209 (44.0%)	217 (45.9%)	184 (40.7%)	151 (33.0%)	150 (32.1%)	

Technical Appendix Table 3. Mental health distress related to H1N1 influenza A/H1N1

Type of distress	Round 1 (n = 503), 19 Aug – 10 Sep 09	Round 2 (n = 481), 25 Sep – 5 Oct 09	Round 3 (n = 475), 27 Oct – 2 Nov 09	Round 4 (n = 473), 28 Nov – 10 Dec 09	Round 5 (n = 452), 28 Jan – 5 Feb 10	Round 6 (n = 457), 7 – 14 Apr 10	Round 7 (n = 467), 24 Jun – 6 Jul 10	p (trend)
Worry about infection								
Worry about yourself becoming infected								
Unlikely / certainly not / unsure	441 (87.7%)	414 (86.1%)	428 (90.1%)	417 (88.3%)	425 (94.0%)	439 (96.3%)	456 (97.6%)	<0.001
Certainly / likely	62 (12.3%)	67 (13.9%)	47 (9.9%)	55 (11.7%)	27 (6.0%)	17 (3.7%)	11 (2.4%)	
Worry that your family being infected								
Unlikely / certainly not / unsure	426 (84.7%)	393 (81.7%)	415 (87.4%)	409 (86.7%)	419 (92.7%)	439 (96.3%)	456 (97.6%)	<0.001
Certainly / likely	77 (15.3%)	88 (18.3%)	60 (12.6%)	63 (13.3%)	33 (7.3%)	17 (3.7%)	11 (2.4%)	
Mental health distress								
Panic much about swine flu								
Unlikely / certainly not / unsure	491 (97.6%)	475 (98.8%)	472 (99.4%)	470 (99.6%)	450 (99.6%)	453 (99.3%)	465 (99.6%)	0.029
Certainly / likely	12 (2.4%)	6 (1.2%)	3 (0.6%)	2 (0.4%)	2 (0.4%)	3 (0.7%)	2 (0.4%)	
Feel much depressed because of swine flu								
Unlikely / certainly not / unsure	491 (97.6%)	477 (99.2%)	473 (99.6%)	471 (99.8%)	450 (99.6%)	453 (99.3%)	465 (99.6%)	0.051
Certainly / likely	12 (2.4%)	4 (0.8%)	2 (0.4%)	1 (0.2%)	2 (0.4%)	3 (0.7%)	2 (0.4%)	
Feel much emotionally disturbed because of swine flu								
Unlikely / certainly not / unsure	489 (97.4%)	472 (98.1%)	469 (98.7%)	471 (99.8%)	450 (99.6%)	453 (99.3%)	464 (99.4%)	0.062
Certainly / likely	13 (2.6%)	9 (1.9%)	6 (1.3%)	1 (0.2%)	2 (0.4%)	3 (0.7%)	3 (0.6%)	
Any of the above								
No	420 (83.7%)	391 (81.3%)	412 (86.7%)	408 (86.3%)	418 (92.5%)	439 (96.1%)	454 (97.2%)	<0.001
Yes	82 (16.3%)	90 (18.7%)	63 (13.3%)	65 (13.7%)	34 (7.5%)	18 (3.9%)	13 (2.8%)	
Mental health distress score due to influenza A/H1N1 (ranged from 1 = Very mild to 10 = Extremely severe)								
<5	319 (63.5%)	275 (57.4%)	286 (60.9%)	282 (59.7%)	265 (58.8%)	294 (64.5%)	346 (74.4%)	<0.001
>5	183 (36.5%)	204 (42.6%)	184 (39.1%)	190 (40.3%)	186 (41.2%)	162 (35.5%)	119 (25.6%)	
<4	238 (47.4%)	209 (43.6%)	237 (50.4%)	218 (46.2%)	190 (42.1%)	230 (50.4%)	286 (61.5%)	
4–7	250 (49.8%)	258 (53.9%)	222 (47.2%)	244 (51.7%)	254 (56.3%)	223 (48.9%)	178 (38.3%)	
>7	14 (2.8%)	12 (2.5%)	11 (2.3%)	10 (2.1%)	7 (1.6%)	3 (0.7%)	1 (0.2%)	
It does not matter much if I contract the human swine flu virus								
Disagree / Don’t know	294 (58.6%)	274 (57.1%)	254 (53.5%)	282 (59.6%)	230 (50.9%)	244 (53.4%)	230 (49.3%)	<0.001
Agree	208 (41.4%)	206 (42.9%)	221 (46.5%)	191 (40.4%)	222 (49.1%)	213 (46.6%)	237 (50.7%)	

Technical Appendix Table 4. Knowledge and misconceptions about the modes of transmission of influenza A/H1N1

Knowledge and misconceptions	Round 1 (n = 503), 19 Aug – 10 Sep 09	Round 2 (n = 481), 25 Sep – 5 Oct 09	Round 3 (n = 475), 27 Oct – 2 Nov 09	Round 4 (n = 473), 28 Nov – 10 Dec 09	Round 5 (n = 452), 28 Jan – 5 Feb 10	Round 6 (n = 457), 7 – 14 Apr 10	Round 7 (n = 467), 24 Jun – 6 Jul 10	*p* (trend)
Correct knowledge about modes of transmission								
Could be transmitted via droplets (e.g., sneeze)	486 (96.6%)	474 (98.5%)	466 (98.1%)	459 (97.0%)	418 (92.5%)	429 (94.1%)	432 (92.5%)	<0.001
Could be transmitted via touching body of infected persons	346 (68.8%)	356 (74.0%)	359 (75.6%)	343 (72.5%)	285 (63.1%)	295 (64.7%)	290 (62.1%)	<0.001
Could be transmitted via touching contaminated objects	338 (67.2%)	334 (69.4%)	344 (72.4%)	333 (70.4%)	233 (51.5%)	245 (53.7%)	312 (66.8%)	<0.001
All above items being correct								
No	242 (48.1%)	200 (41.6%)	175 (36.8%)	200 (42.3%)	290 (64.2%)	261 (57.1%)	247 (52.9%)	<0.001
Yes	261 (51.9%)	281 (58.4%)	300 (63.2%)	273 (57.7%)	162 (35.8%)	196 (42.9%)	22.0 (47.1%)	
Misconceptions about modes of transmission								

Knowledge and misconceptions	Round 1 (n = 503), 19 Aug – 10 Sep 09	Round 2 (n = 481), 25 Sep – 5 Oct 09	Round 3 (n = 475), 27 Oct – 2 Nov 09	Round 4 (n = 473), 28 Nov – 10 Dec 09	Round 5 (n = 452), 28 Jan – 5 Feb 10	Round 6 (n = 457), 7 – 14 Apr 10	Round 7 (n = 467), 24 Jun – 6 Jul 10	*p* (trend)
Could be transmitted via airborne with a long distance (from one building to another one)	162 (32.3%)	147 (30.6%)	151 (31.8%)	108 (22.8%)	107 (23.7%)	90 (19.7%)	107 (22.9%)	<0.001
Could be transmitted via insect bites	96 (19.1%)	81 (16.9%)	69 (14.5%)	79 (16.7%)	93 (20.6%)	86 (18.9%)	122 (26.1%)	<0.001
Could be transmitted via water sources (e.g., reservoirs)	125 (24.9%)	97 (20.2%)	108 (22.7%)	93 (19.7%)	119 (26.3%)	113 (24.8%)	161 (34.5%)	<0.001
Could be transmitted via well-cooked pork	12 (2.4%)	7 (1.5%)	10 (2.1%)	17 (3.6%)	16 (3.5%)	18 (3.9%)	29 (6.2%)	<0.001
Any one of above								
No	251 (49.9%)	258 (53.6%)	239 (50.3%)	293 (61.9%)	232 (51.3%)	256 (56.0%)	224 (48.0%)	0.578
Yes	252 (50.1%)	223 (46.4%)	236 (49.7%)	180 (38.1%)	220 (48.7%)	201 (44.0%)	243 (52.0%)	

Technical Appendix Table 5. Perceived severity of influenza A/H1N1

Perceived severity	Round 1 (n = 503), 19 Aug – 10 Sep 09	Round 2 (n = 481), 25 Sep – 5 Oct 09	Round 3 (n = 475), 27 Oct – 2 Nov 09	Round 4 (n = 473), 28 Nov – 10 Dec 09	Round 5 (n = 452), 28 Jan – 5 Feb 10	Round 6 (n = 457), 7 – 14 Apr 10	Round 7 (n = 467), 24 Jun – 6 Jul 10	*p* (trend)
Mortality rate for adults								
<1% / Don't know	345 (68.6%)	291 (60.6%)	278 (58.5%)	254 (53.7%)	237 (52.4%)	300 (65.6%)	351 (75.2%)	<0.001
≥1%	158 (31.4%)	189 (39.4%)	197 (41.5%)	219 (46.3%)	215 (47.6%)	157 (34.4%)	116 (24.8%)	
Would cause severe irreversible body damages								
Disagree / Don't know	418 (83.1%)	399 (83.0%)	388 (81.7%)	364 (77.0%)	330 (73.0%)	350 (76.6%)	350 (74.9%)	<0.001
Agree	85 (16.9%)	82 (17.0%)	87 (18.3%)	109 (23.0%)	122 (27.0%)	107 (23.4%)	117 (25.1%)	
What would you think in the coming year, the effects of H1N1 influenza virus on the infected person would become milder, more harmful, or similar to the current situation?								
Milder / Similar to current situation / the same	410 (85.6%)	390 (84.1%)	410 (88.7%)	358 (78.3%)	401 (89.5%)	428 (95.3%)	440 (94.8%)	<0.001
More harmful	69 (14.4%)	74 (15.9%)	52 (11.3%)	99 (21.7%)	47 (10.5%)	21 (4.7%)	24 (5.2%)	
Perceived severity of H1N1 compared to seasonal flu								
Mortality rate								
Much lower / a little lower / the same	275 (55.7%)	228 (47.8%)	210 (44.5%)	203 (43.3%)	174 (38.8%)	184 (40.7%)	197 (42.7%)	<0.001
A bit higher / much higher	219 (44.3%)	249 (52.2%)	262 (55.5%)	266 (56.7%)	274 (61.2%)	268 (59.3%)	264 (57.3%)	
Infectivity rate								
Much lower / a little lower / the same	315 (63.3%)	316 (66.0%)	291 (61.4%)	277 (58.8%)	308 (68.3%)	274 (60.4%)	311 (66.6%)	0.374
A bit higher / much higher	183 (36.7%)	163 (34.0%)	183 (38.6%)	194 (41.2%)	143 (31.7%)	180 (39.6%)	156 (33.4%)	
Severity of body damages								
Much lower / a little lower / the same	264 (53.8%)	227 (48.0%)	211 (44.6%)	176 (37.4%)	147 (32.9%)	172 (38.0%)	175 (37.6%)	<0.001
A bit higher / much higher	227 (46.2%)	246 (52.0%)	262 (55.4%)	294 (62.6%)	300 (67.1%)	281 (62.0%)	290 (62.4%)	
Perceived severity of H1N1 compared to SARS								
Mortality rate								
Lower / the same / don't know	492 (97.8%)	473 (98.3%)	471 (99.2%)	464 (98.1%)	443 (98.0%)	451 (98.7%)	460 (98.5%)	0.390
Higher	11 (2.2%)	8 (1.7%)	4 (0.8%)	9 (1.9%)	9 (2.0%)	6 (1.3%)	7 (1.5%)	
Infectivity rate								
Lower / the same / don't know	415 (83.0%)	370 (77.2%)	389 (82.1%)	400 (84.6%)	397 (87.8%)	422 (92.3%)	444 (95.1%)	<0.001
Higher	85 (17.0%)	109 (22.8%)	85 (17.9%)	73 (15.4%)	55 (12.2%)	35 (7.7%)	23 (4.9%)	

Technical Appendix Table 6. Perceptions related to susceptibility and community outbreak of influenza A/H1N1

Perceived susceptibility	Round 1 (n = 503), 19 Aug – 10 Sep 09	Round 2 (n = 481), 25 Sep – 5 Oct 09	Round 3 (n = 475), 27 Oct – 2 Nov 09	Round 4 (n = 473), 28 Nov – 10 Dec 09	Round 5 (n = 452), 28 Jan – 5 Feb 10	Round 6 (n = 457), 7 – 14 Apr 10	Round 7 (n = 467), 24 Jun – 6 Jul 10	*p* (trend)
Perceived high or very high chance of contracting Flu A (H1N1) in the coming year								
(a) The respondent	77 (16.6%)	116 (25.4%)	78 (17.3%)	66 (14.7%)	52 (11.9%)	34 (7.7%)	12 (2.6%)	<0.001
(b) Family members	69 (14.9%)	114 (25.2%)	75 (16.7%)	68 (15.2%)	48 (11.0%)	34 (7.7%)	12 (2.6%)	<0.001
(c) The general public	90 (20.9%)	133 (31.4%)	79 (20.3%)	73 (17.0%)	58 (14.0%)	34 (8.0%)	12 (2.6%)	<0.001
Feel confident that you and your family are and will be free from infection								
Unlikely / certainly not / unsure	126 (25.0%)	137 (28.6%)	117 (24.7%)	97 (20.6%)	111 (24.6%)	68 (14.9%)	36 (7.7%)	<0.001
Certainly / likely	377 (75.0%)	342 (71.4%)	357 (75.3%)	373 (79.4%)	341 (75.4%)	388 (85.1%)	430 (92.3%)	
Perception related to community outbreak of H1N1								
Perceived chance of having a large-scale local A/H1N1 outbreak in Hong Kong in the coming year								
Unlikely / most unlikely / certainly not / unsure	295 (58.6%)	247 (51.4%)	311 (65.6%)	404 (85.4%)	401 (88.7%)	429 (94.3%)	451 (96.6%)	<0.001
Certainly / most likely / likely	208 (41.4%)	234 (48.6%)	163 (34.4%)	69 (14.6%)	51 (11.3%)	26 (5.7%)	16 (3.4%)	
Perceived duration of H1N1 flu A epidemic								
$\leq$12 mo	321 (67.6%)	296 (63.2%)	293 (63.7%)	266 (57.8%)	270 (61.8%)	265 (59.6%)	347 (76.9%)	0.002
>12 mo	154 (32.4%)	172 (36.8%)	167 (36.3%)	194 (42.2%)	167 (38.2%)	180 (40.4%)	104 (23.1%)	
Perceived number of death cases in the coming year								
$\leq$50	432 (90.2%)	379 (82.8%)	363 (78.9%)	401 (86.1%)	381 (86.8%)	428 (95.1%)	458 (99.1%)	<0.001
>50	47 (9.8%)	79 (17.2%)	97 (21.1%)	65 (13.9%)	58 (13.2%)	22 (4.9%)	4 (0.9%)	
Perceived number of hidden cases in the community								
Very few / a few	217 (44.7%)	150 (32.3%)	202 (43.8%)	226 (49.1%)	205 (47.1%)	215 (49.4%)	337 (73.6%)	<0.001
Quite a lot / many	268 (55.3%)	315 (67.7%)	259 (56.2%)	234 (50.9%)	230 (52.9%)	220 (50.6%)	121 (26.4%)	

Technical Appendix Table 7. Perceived efficacy of measures for preventing influenza A/H1N1

Perceived efficacy	Round 1 (n = 503), 19 Aug – 10 Sep 09	Round 2 (n = 481), 25 Sep – 5 Oct 09	Round 3 (n = 475), 27 Oct – 2 Nov 09	Round 4 (n = 473), 28 Nov – 10 Dec 09	Round 5 (n = 452), 28 Jan – 5 Feb 10	Round 6 (n = 457), 7 – 14 Apr 10	Round 7 (n = 467), 24 Jun – 6 Jul 10	*p* (trend)
Wearing face masks in public areas								
Not effective at all / Not very effective	47 (9.4%)	31 (6.4%)	49 (10.3%)	41 (8.7%)	39 (8.6%)	39 (8.5%)	41 (8.8%)	0.940
Quite effective / Very effective	454 (90.6%)	450 (93.6%)	426 (89.7%)	432 (91.3%)	413 (91.4%)	418 (91.5%)	426 (91.2%)	
Washing hands frequently								
Not effective at all / Not very effective	3 (0.6%)	4 (0.8%)	3 (0.6%)	6 (1.3%)	7 (1.5%)	6 (1.3%)	7 (1.5%)	0.071
Quite effective / Very effective	499 (99.4%)	477 (99.2%)	472 (99.4%)	467 (98.7%)	445 (98.5%)	451 (98.7%)	460 (98.5%)	
Injection of seasonal flu vaccine								
Not effective at all / Not very effective	268 (55.0%)	266 (57.2%)	264 (57.5%)	267 (58.4%)	286 (64.9%)	286 (63.8%)	290 (63.5%)	<0.001
Quite effective / Very effective	219 (45.0%)	199 (42.8%)	195 (42.5%)	190 (41.6%)	155 (35.1%)	162 (36.2%)	167 (36.5%)	
Injection of flu A (H1N1) vaccine								
Not effective at all / Not very effective	50 (10.7%)	44 (9.9%)	32 (7.4%)	49 (11.3%)	72 (16.9%)	68 (15.7%)	58 (12.8%)	0.007
Quite effective / Very effective	416 (89.3%)	401 (90.1%)	399 (92.6%)	385 (88.7%)	354 (83.1%)	365 (84.3%)	395 (87.2%)	
Taking traditional Chinese medicine								
Not effective at all / Not very effective	186 (41.9%)	172 (42.8%)	166 (42.6%)	178 (44.0%)	146 (37.5%)	175 (42.2%)	157 (38.1%)	0.253
Quite effective / Very effective	258 (58.1%)	230 (57.2%)	224 (57.4%)	227 (56.0%)	243 (62.5%)	240 (57.8%)	255 (61.9%)	
Avoid going to crowded places								
Not effective at all / Not very effective	24 (4.8%)	18 (3.7%)	20 (4.2%)	30 (6.3%)	29 (6.4%)	26 (5.7%)	22 (4.7%)	0.523
Quite effective / Very effective	479 (95.2%)	463 (96.3%)	455 (95.8%)	443 (93.7%)	423 (93.6%)	431 (94.3%)	445 (95.3%)	

Technical Appendix Table 8. Evaluation of government preparedness and performance toward influenza A/H1N1

Evaluation	Round 1 (n = 503), 19 Aug – 10 Sep 09	Round 2 (n = 481), 25 Sep – 5 Oct 09	Round 3 (n = 475), 27 Oct – 2 Nov 09	Round 4 (n = 473), 28 Nov – 10 Dec 09	Round 5 (n = 452), 28 Jan – 5 Feb 10	Round 6 (n = 457), 7 – 14 Apr 10	Round 7 (n = 467), 24 Jun – 6 Jul 10	*p* (trend)
Evaluation on government performance								
Timeliness of prevention measures	6.1 (1.6)	6.2 (1.4)	6.2 (1.4)	6.2 (1.4)	6.0 (1.4)	6.3 (1.4)	6.2 (1.5)	
Effectiveness of prevention measures	6.0 (1.7)	6.1 (1.4)	6.2 (1.3)	6.2 (1.4)	5.9 (1.5)	6.1 (1.5)	6.2 (1.6)	
Explaining clearly to general public	6.2 (1.7)	6.3 (1.5)	6.2 (1.4)	6.2 (1.5)	5.9 (1.6)	6.1 (1.6)	6.1 (1.6)	
Adequacy of quarantine and disinfection procedure	6.2 (1.7)	6.3 (1.4)	6.2 (1.3)	6.2 (1.4)	6.1 (1.5)	6.2 (1.5)	6.1 (1.7)	
Collaboration between governmental departments	5.6 (1.8)	5.8 (1.6)	5.7 (1.4)	5.8 (1.5)	5.6 (1.5)	5.7 (1.7)	5.7 (1.6)	
General evaluation	6.0 (1.6)	6.2 (1.3)	6.2 (1.3)	6.2 (1.3)	6.0 (1.4)	6.2 (1.5)	6.3 (1.5)	
Average score	6.0 (1.5)	6.2 (1.3)	6.1 (1.2)	6.1 (1.3)	5.9 (1.4)	6.1 (1.4)	6.1 (1.5)	
Average score <5	100 (20.7%)	62 (13.1%)	55 (11.9%)	65 (14.0%)	91 (20.6%)	75 (16.7%)	88 (19.0%)	0.101
The HK Government is overreacting with its current preventive measures								
Not overreacting at all / Not overreacting /Just right	469 (93.4%)	451 (94.2%)	460 (97.0%)	462 (98.1%)	409 (90.7%)	425 (93.0%)	430 (92.1%)	0.143
Overreacting somewhat / extremely overreacting	33 (6.6%)	28 (5.8%)	14 (3.0%)	9 (1.9%)	42 (9.3%)	32 (7.0%)	37 (7.9%)	
Evaluation on government preparedness								
Hong Kong will not have enough vaccine for human swine flu								
Disagree / Don’t know	322 (64.0%)	333 (69.2%)	351 (73.9%)	378 (79.9%)	377 (83.4%)	381 (83.4%)	445 (95.3%)	<0.001
Agree	181 (36.0%)	148 (30.8%)	124 (26.1%)	95 (20.1%)	75 (16.6%)	76 (16.6%)	22 (4.7%)	
Hong Kong will not have enough medication for human swine flu								
Disagree / Don’t know	384 (76.5%)	397 (82.5%)	384 (80.8%)	395 (83.5%)	371 (82.1%)	377 (82.5%)	450 (96.4%)	<0.001
Agree	118 (23.5%)	84 (17.5%)	91 (19.2%)	78 (16.5%)	81 (17.9%)	80 (17.5%)	17 (3.6%)	
HK will be able to control the Flu A (H1N1) epidemic								
Disagree / Don’t know	115 (22.9%)	127 (26.4%)	85 (17.9%)	76 (16.1%)	61 (13.5%)	45 (9.8%)	24 (5.1%)	<0.001
Agree	388 (77.1%)	354 (73.6%)	390 (82.1%)	397 (83.9%)	391 (86.5%)	412 (90.2%)	443 (94.9%)	

Technical Appendix Table 9. Perceptions on policy-related issues for influenza A/H1N1

Perception	Round 1 (n = 503), 19 Aug – 10 Sep 09	Round 2 (n = 481), 25 Sep – 5 Oct 09	Round 3 (n = 475), 27 Oct – 2 Nov 09	Round 4 (n = 473), 28 Nov – 10 Dec 09	Round 5 (n = 452), 28 Jan – 5 Feb 10	Round 6 (n = 457), 7 – 14 Apr 10	Round 7 (n = 467), 24 Jun – 6 Jul 10	*p* (trend)
It is not necessary to have class suspension for kindergartens/ primary schools, even there are infected cases found in the schools								
Disagree / Don’t know	–	399 (83.0%)	338 (71.2%)	332 (70.2%)	282 (62.4%)	312 (68.3%)	245 (52.5%)	<0.001
Agree	–	82 (17.0%)	137 (28.8%)	141 (29.8%)	170 (37.6%)	145 (31.7%)	222 (47.5%)	
It is not necessary to have class suspension for secondary schools, even there are infected cases found in the schools								
Disagree / Don’t know	–	301 (62.6%)	205 (43.2%)	217 (45.9%)	166 (36.7%)	166 (36.3%)	108 (23.1%)	<0.001
Agree	–	180 (37.4%)	270 (56.8%)	256 (54.1%)	286 (63.3%)	291 (63.7%)	359 (76.9%)	
The Hong Kong government should cancel all pandemic control measures for H1N1 influenza A								
Disagree / Don’t know	–	464 (96.7%)	461 (97.1%)	453 (95.8%)	437 (96.7%)	436 (95.4%)	394 (84.4%)	<0.001
Agree	–	16 (3.3%)	14 (2.9%)	20 (4.2%)	15 (3.3%)	21 (4.6%)	73 (15.6%)	
The Hong Kong government should suspend all pandemic control measures for H1N1 influenza A								
Disagree / Don’t know	–	–	–	–	330 (73.0%)	376 (82.3%)	263 (56.3%)	<0.001
Agree	–	–	–	–	122 (27.0%)	81 (17.7%)	204 (43.7%)	
The government should treat human swine flu in the same manner as the seasonal flu								
Disagree / Unsure	236 (46.9%)	249 (52.0%)	242 (50.9%)	265 (56.0%)	276 (61.1%)	220 (48.1%)	213 (45.6%)	0.225
Agree	267 (53.1%)	230 (48.0%)	233 (49.1%)	208 (44.0%)	176 (38.9%)	237 (51.9%)	254 (54.4%)	
Do you think you understand clearly the preventive measures and treatment strategies/policies advocated by the HK Government								
Not clear at all / not quite clear	205 (40.8%)	190 (39.6%)	205 (43.2%)	215 (45.5%)	159 (35.2%)	214 (47.0%)	242 (51.8%)	<0.001
Quite clear / very clear	298 (59.2%)	290 (60.4%)	270 (56.8%)	258 (54.5%)	293 (64.8%)	241 (53.0%)	225 (48.2%)	
